# Nlrx1-Regulated Defense and Metabolic Responses to *Aspergillus fumigatus* Are Morphotype and Cell Type Specific

**DOI:** 10.3389/fimmu.2021.749504

**Published:** 2021-11-01

**Authors:** Bridget Kastelberg, Tariq Ayubi, Nuria Tubau-Juni, Andrew Leber, Raquel Hontecillas, Josep Bassaganya-Riera, Shiv D. Kale

**Affiliations:** NIMML Institute, Blacksburg, VA, United States

**Keywords:** NLRX1, aspergillus fumigalus, glycolysis, nod-like proteins, defense, fungi, reactive oxygen species, NADPH oxidase

## Abstract

The Nlr family member X1 (Nlrx1) is an immuno-metabolic hub involved in mediating effective responses to virus, bacteria, fungi, cancer, and auto-immune diseases. We have previously shown that Nlrx1 is a critical regulator of immune signaling and mortality in several models of pulmonary fungal infection using the clinically relevant fungus *Aspergillus fumigatus*. In the absence of Nlrx1, hosts produce an enhanced Th2 response primarily by CD103+ dendritic cell populations resulting in enhanced mortality *via* immunopathogenesis as well as enhanced fungal burden. Here, we present our subsequent efforts showcasing loss of Nlrx1 resulting in a decreased ability of host cells to process *A. fumigatus* conidia in a cell-type-specific manner by BEAS-2B airway epithelial cells, alveolar macrophages, bone marrow-derived macrophages, but not bone marrow-derived neutrophils. Furthermore, loss of Nlrx1 results in a diminished ability to generate superoxide and/or generic reactive oxygen species during specific responses to fungal PAMPs, conidia, and hyphae. Analysis of glycolysis and mitochondrial function suggests that Nlrx1 is needed to appropriately shut down glycolysis in response to *A. fumigatus* conidia and increase glycolysis in response to hyphae in BEAS-2B cells. Blocking glycolysis and pentose phosphate pathway (PPP) *via* 2-DG and NADPH production through glucose-6-phosphate dehydrogenase inhibitor resulted in significantly diminished conidial processing in wild-type BEAS-2B cells to the levels of Nlrx1-deficient BEAS-2B cells. Our findings suggest a need for airway epithelial cells to generate NADPH for reactive oxygen species production in response to conidia *via* PPP. In context to fungal pulmonary infections, our results show that Nlrx1 plays significant roles in host defense *via* PPP modulation of several aspects of metabolism, particularly glycolysis, to facilitate conidia processing in addition to its critical role in regulating immune signaling.

## Introduction

*Aspergillus fumigatus* is a clinically relevant, fungal pathogen that causes a wide variety of pulmonary disease manifestations. As an opportunistic pathogen, *A. fumigatus* relies on inherent deficiencies in the host immune system to facilitate disease (1). These clinically identified defects are often associated with an inability by the host to appropriately recruit innate immune cells, primarily macrophages and neutrophils, in order to identify, process, and clear the fungus (1). While much focus has been placed on macrophages and neutrophils, the importance of dendritic cell subsets, airway epithelial cells, and T cells as orchestrators of the innate and adaptive immune system has become evident (2–7). There has also been substantial effort to understand intra- and intercellular communication within and among host cell populations that facilitates these processes. Our prior work identified the host nod-like receptor X1 (Nlrx1) as a critical regulator of defense and immune signaling that functioned in a cell- and context-specific manner (6). Here, we expand on aspects of that work using a variety of *in vitro* cell models and Nlrx1 knockouts to unravel novel aspects of defense responses and metabolism that are fungal morphotype, host cell type, and Nlrx1 dependent.

Studies to dissect defense responses to *A. fumigatus* have been primarily pursued in macrophages, neutrophils, and airway epithelial cells. Macrophages rapidly ingest *A. fumigatus* resting or swollen conidia *via* receptor-mediated phagocytosis followed by phagosome acidification and reactive oxygen species production (8–11). This process has been shown to be dependent upon LC3-associated phagocytosis, NOX2 NADPH oxidase complex, and enhancement of glycolysis (8–10). Neutrophils have also been shown to rapidly internalize and produce superoxide, hydrogen peroxide, and hypochlorous acid in response to resting and swollen conidia; however, the relative production of these defense compounds was significantly lower in resting conidia in comparison to swollen (12). Further non-oxidative responses have also been observed (13). Responses to the hyphal form of *A. fumigatus* results in the production of neutrophil extracellular traps that are thought to ensnare the fungus as well as allow for the generation of massive levels of reactive oxygen species *via* NADPH oxidases and myeloperoxidase (13, 14). Both neutrophils and macrophages provide important examples of morphotype-specific responses by critical host cell populations (13, 15).

*In vitro* internalization rates of *A. fumigatus* conidia by airway epithelial cells are highly variable, ranging from 10%–50%, and dependent on cell type (BEAS-2B, 16HBE14o-, A549, primary bronchial, nasal, etc.) [Reviewed in (16)]. Recognition of conidia by airway epithelial cells occurs in at least a Tlr2 manner and involves the increased expression of Dectin-1 (17). Processing of conidia has been shown to be dependent on host phosphoinositide 3-kinase, flotillin-2, caveolin, and Rab5c (11). Further several putative defensins have been identified to be expressed in response to conidia (17). A growing body of literature indicates that conidia are also processed extracellularly or made inactive though the precise mechanism(s) remaining unknown (11, 18). One study using transmission electron microscopy did not observe internalization of *A. fumigatus* conidia by airway epithelial cells post challenge in immunosuppressed mice, but rather found conidia at junction sites between ciliated and/or goblet cells (19). Preliminary data on the generation of reactive oxygen species have been observed by airway epithelial cells after prolonged exposure to viable *A. fumigatus* resting conidia and is dependent on Dectin-1; however, this mechanism also remains unknown (17).

Nlrx1 is a nod-like receptor consisting of a mitochondrial targeting signal, a nucleotide binding domain, and leucine-rich repeat domain. Nlrx1 has been shown to be a critical regulator of host immune and defense responses to a variety of microbial pathogens, cancers, and auto-immune diseases [reviewed recently in (20)]. Mechanistically, Nlrx1 appears to function as a multi-faceted hub. A subset of Nlrx1’s functions include its ability to bind TRAF6/2, thereby mitigating a variety of immune signal transduction pathways (21), interact within the inner mitochondrial membrane, thereby impacting mitochondrial function as well as forms of reactive oxygen species production (22–25), bind TUFM to facilitate autophagy and LC3-associated phagocytosis (26), and insulate the DNA sensing adaptors STING and/or MAVS to mitigate innate immune signaling to a variety of viruses (27, 28). This diversity of concurrent functions has made Nlrx1 a promising therapeutic target for a variety of diseases (29, 30).

Our prior work on *Nlrx1* identified the gene to be differentially expressed during models of invasive pulmonary aspergillosis (31). *In vivo* studies involving several models of invasive pulmonary aspergillosis using *Nlrx1* knockout mice indicated enhanced fungal burden, mortality, and enhanced immune signaling towards a detrimental Th2 response *via* CD103+ dendritic cells in the absence of *Nlrx1* that ultimately resulted in immunopathogenesis-driven mortality (6). We hypothesized that the enhanced fungal burden may function as a double detriment to the Th2-mediated immunopathogenesis. Here, we follow up on our prior observation of enhanced fungal burden by determining if the loss of *Nlrx1* results in enhanced conidial survival in a variety of cell types as well as dissecting how Nlrx1 mediates these processes. Results from this study provided novel insight into how Nlrx1 regulates defense and metabolic responses to *A. fumigatus* in a morphotype- and cell-type-specific manner.

## Materials and Methods

### Cell Extraction, Culture, and Maintenance

*A. fumigatus* strain Af293 was routinely cultured on glucose minimal media at 37°C for 5–7 days. Conidia were harvested in PBS + Tween 20 (0.1%) and passed through a 90-micron filter to remove hyphae and particulate prior to challenge. Fresh aliquots of *A. fumigatus* were taken after every fourth passage. Wild-type and Nlrx1-deficient cells generated by CRISPR-Cas9 (ΔNlrx1) BEAS-2B cells were routinely cultured at 37°C in the presence of 5% CO_2_ using RPMI base medium supplemented with 10% fetal bovine serum and 1× penicillin-streptomycin (6). Wild-type and ΔNlrx1 BEAS-2B cells were grown to a maximum of 75% confluence and discarded after their ninth passage. Wild-type and Nlrx1 knockout (*Nlrx1-/-*, derived from homozygous mice) bone marrow-derived macrophages (BMDMs) and bone marrow-derived neutrophils (BMDNs) were extracted from male mice between 15 and 30 weeks of age as previously described in (32). BMDNs were immediately utilized post purification. BMDMs were differentiated using 25 ng/ml M-CSF. On day 6 post incubation, BMDMs were seeded at 5 × 10^5^ or 5 × 10^4^ cells into 24- and 96-well plates, respectively, and utilized the following day for challenge experiments.

### Challenge Assays

Freshly harvested Af293 conidia were challenged against wild-type and Nlrx1-deficient BMDMs, BMDNs, alveolar macrophages, and immortalized human bronchial epithelial BEAS-2B cells (5 × 10^4^ 96-well, 5 × 10^5^ 24-well, American Type Culture Collection) for 3, 6, 9, 12, and/or 24 h at 37°C in the presence of 5% CO_2_.

For the FUN-1 assay, conidia and mammalian cells were incubated with calcofluor white M2R (CFWM2R, 25 μM) for 15 min to label conidia. Cells and conidia were incubated in NP-40 buffer (250 μl) plus the metabolic dye FUN-1 (2.5 μM) for 1 h at 37°C. Conidia (50,000–60,000) from independent challenges were analyzed by flow cytometry for FUN-1 and CRWM2R fluorescence. Positive gating was determined using conidia challenged onto empty tissue culture wells containing respective cell culture media. Independent experiments were run in triplicate. All experiments were independently repeated. Statistical difference was determined using the Kruskal–Wallis test followed by a Dunn’s test for multiple comparisons. For challenge assays involving inhibitors, BEAS-2B cells were incubated for 3 h with the respective inhibitor prior to challenge and then washed 3× to remove the inhibitor.

For the XTT cell viability assay (CyQuant), cells (5 × 10^4^, 96-well) were challenged for 24 h with *A. fumigatus* conidia, washed with PBS, and resuspended in fresh media (100 μl). Cells were then vigorously pipetted to lyse BEAS-2B cells as viable BEAS-2B cells masked XTT metabolism by the fungus. No PBS wash or vigorous pipetting was required for alveolar macrophage and BMDM challenges. XTT solution was made fresh as per the manufacturer’s instruction and 50 μl was added to each well. Cells were incubated for 2–4 h at 37°C and the absorbance was read at 450 and 660 nm (background correction). Corrected absorbance from media only wells was subtracted from challenges to determine specific absorbance. All experiments were independently repeated. Statistical significance was determined by Kruskal–Wallis test followed by a Dunn’s test for multiple comparisons.

For the colony-forming unit assay, conidia (5 × 10^3^) were challenged against cells (5 × 10^4^) for 12 h and were resuspended in 250 μl of NP-40 buffer, plated (50 μl) onto GMM plates, and incubated for 12–24 h at 37°C. All experiments were independently repeated. Statistical significance was determined by the Kruskal–Wallis test followed by a Dunn’s test for multiple comparisons.

### Measurement of Reactive Oxygen Species Production and Oxidative Stress

Wild-type and Nlrx1-deficient BEAS-2B airway epithelial cells, BMDMs, and BMDNs (50,000 per well, 96-well plate format) were pre-stained with ROS- and OS-specific fluorescent dyes (Enzo scientific, ROS-ID**^®^** ROS/RNS detection kit) for 30 min prior to challenge with fungal pathogen-associated molecular patterns (PAMPs) curdlan (100 ng/ml, InvivoGen), zymosan depleted (100 ng/ml, InvivoGen), zymosan (100 ng/ml, InvivoGen), killed hyphae (100 ng/ml, laboratory derived), and killed Af293 conidia (50,000 per well in 10 μl). Cells were incubated at 37°C in the presence of 5% CO_2_ prior to and post challenge. Fluorescence was measured for ROS production (Excitation: 490/14, Emission: 525/20) and OS production (Excitation: 550/20, Emission: 620/20) at 30, 60, 90, 180, 360, and 540 min post challenge using a Cytation 5 plate reader at 37°C in the presence of 5% CO_2_. Experiments were conducted at 6× and repeated independently. Statistical difference was determined using the Kruskal–Wallis test followed by a Dunn’s test for multiple comparisons.

### Modified Mitochondrial and Glycolytic Stress Tests

Both mitochondrial and glycolytic stress tests were performed as described by the manufacturer’s protocol (Agilent). Cells were plated at a density of 50,000 cells per well (24-well format) approximately 24 h prior to run. Cells were washed three times with PBS (500 μl) and incubated for 1 h in serum and bicarbonate free DMEM in the absence of glutamine (glycolytic) or in the presence of 1 mM glutamine (mitochondrial) at 37°C. After initial baseline equilibration, fungal PAMPs curdlan (100 ng/ml) and zymosan depleted (100 ng/ml), killed conidia (50,000), killed hyphae (100 ng/ml), and control media were injected *via* port-A and allowed to mix prior to sequential addition of compounds for glycolytic and mitochondrial stress tests. For the mitochondrial stress test, basal respiration was determined by averaging the oxygen consumption rate (OCR) prior to treatment with control media. ATP production was determined by measuring the minimal OCR post oligomycin treatment and the average basal OCR. Maximal respiration was calculated based on the difference in maximal OCR post FCCP treatment and minimal OCR post oligomycin treatment. Spare capacity was determined by the difference in average basal OCR and the maximal OCR post FCCP treatment. For the glycolytic stress test, glycolytic activity was determined by the difference in basal extracellular acidification rate (ECAR) and ECAR post glucose treatment. Glycolytic capacity was determined by the difference in basal ECAR and ECAR post oligomycin treatment. Glycolytic reserve was determined by the difference in the ECAR post glucose injection and the ECAR post 2-DG injection. All experiments were independently repeated. *N* = 6. Statistical significance was assessed by the Kruskal–Wallis test followed by a Dunn’s test for multiple comparisons.

## Results

**Figure 1:**
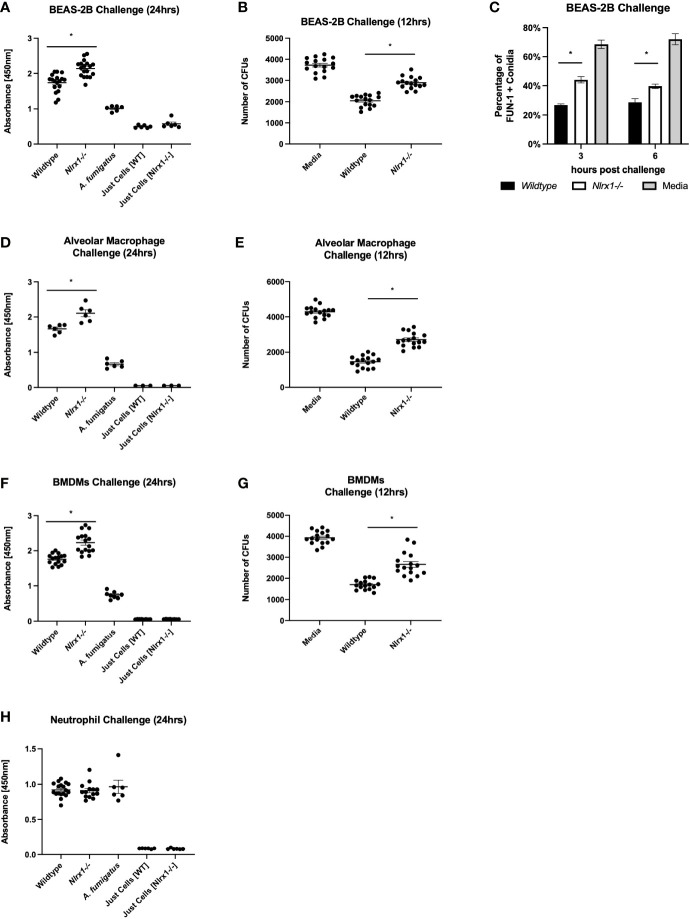
Nlrx1 mediated *A. fumigatus* processing occurs in a cell-type-specific manner. Viable *A. fumigatus* conidia were challenged against wild-type and Nlrx1-deficient **(A–C)** BEAS-2B airway epithelial cells, **(F, G)** bone marrow-derived macrophages, **(D, E)** alveolar macrophages, and **(H)** bone marrow-derived neutrophils. Conidial viability was assessed *via* **(A, D, F, H)** XTT viability assay 24 h post challenge, **(B, E, G)** the colony-forming unit assay 12 h post challenge, and **(C)** FUN-1+ fluorescence assay 3 h post challenge. *p* < 0.05, indicated by asterisk *via* Kruskal–Wallis test followed by a Dunn’s test for multiple comparisons.

We initially set out to determine if *Nlrx1* contributes to the host cell’s ability to process *A. fumigatus* conidia. Our prior efforts had indicated that loss of *Nlrx1* resulted in enhanced fungal burden in immuno-competent and suppressed mice when inoculated with *A. fumigatus* (6). Loss of *Nlrx1* in BEAS-2B airway epithelial cells, alveolar macrophages, and BMDMs, but not BMDNs resulted in significantly decreased conidial processing using both colony-forming units 12 h post challenge as well as an XTT metabolism 24 h post challenge (*p* < 0.05 where denoted by an asterisk) (**Figures 1A, B, D–H**). We had previously established a flow cytometry assay to measure conidial viability through FUN-1 fluorescence at early time points post challenge (11). This assay also indicated that Nlrx1-deficient BEAS-2B (ΔNlrx1) cells processed approximately half as many viable conidia as wild-type cells 3 and 6 h post challenge (*p* < 0.05) (**Figure 1C**). Our *in vitro* results suggest that the contributions of Nlrx1 to fungal processing are relevant to airway epithelial cell and macrophages, two critical cell populations involved in early response to and processing of *A. fumigatus*. BMDNs did not have an altered ability to process conidia. This finding is quite relevant as enhanced mortality and fungal burden dependent on Nlrx1 were greater in *in vivo* models of IPA where neutrophils were depleted or rendered ineffectual (6). It has yet to be determined if Nlrx1 impacts processing of hyphae in these cell types.

**Figure 2:**
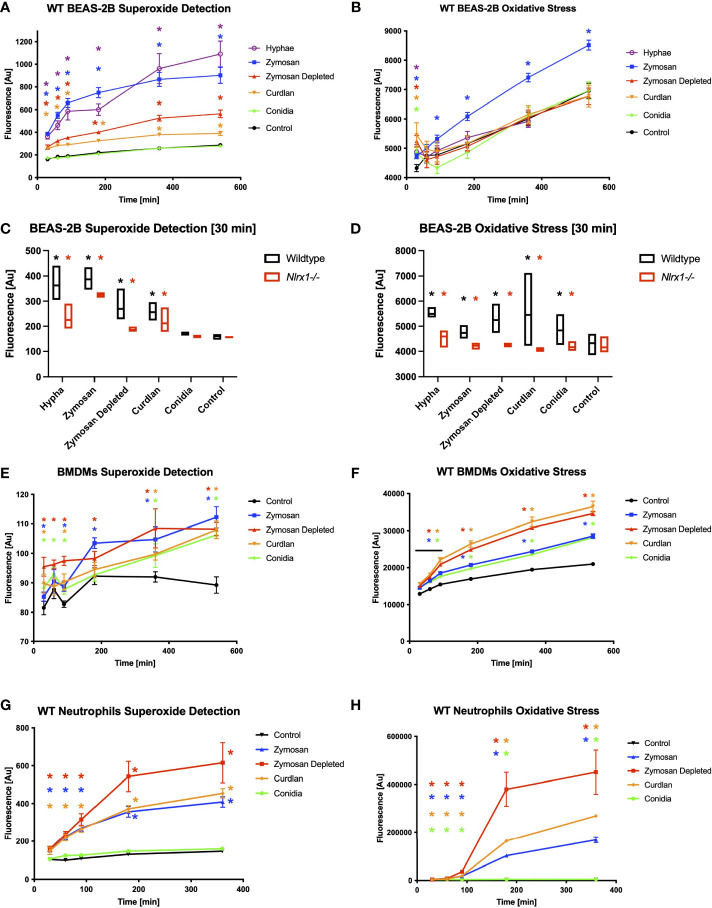
Reactive oxygen species production and superoxide production in response to *A. fumigatus* conidia hyphae, and a subset of fungal PAMPs is dependent on Nlrx1. BEAS-2B airway epithelial cells were pre-stained for 30 min with cell-permeable, stable fluorescent dyes specific for **(A, C, E, G)** superoxide production or **(B, D, F, G)** general reactive oxygen species production prior to challenge. **(A, B)** Wild-type BEAS-2B cells, **(E, F)** bone marrow-derived macrophages, and **(G, H)** neutrophils were challenged for 9 h with killed *A. fumigatus* conidia, hyphae, and fungal PAMPs, and fluorescence was measured 30, 60, 90, 180, 360, and 540 min post challenge. Colored asterisk (corresponding to treatment group) denotes *p* < 0.05 *via* Kruskal–Wallis test followed by a Dunn’s test for multiple comparisons against the control. **(C, D)** Fluorescence emission from wild-type and Nlrx1-deficient BEAS-2B cells 30 min post challenge. Black asterisk denotes *p* < 0.05 *via* Kruskal–Wallis test followed by a Dunn’s test for multiple comparisons against the control. Red asterisk denotes *p* < 0.05 between genotypes for a given treatment group. **Supplementary Figures S1**–**S3** show time course data for Nlrx1-deficient background compared to wild type.

Prior work of others indicates reactive oxygen species production, specifically superoxide produced by NADH and NADPH oxidases, are critical for processing *A. fumigatus* conidia by macrophages (8–10). Nlrx1 has been shown to be needed for robust generation of reactive oxygen species to combat a variety of microbial pathogens (22–25). We sought to determine if Nlrx1 regulated the production of general oxidative stress (ROS) as well as specific generation of superoxide $\left({\text{O}}_{2}^{-}\right)$ in response to *A. fumigatus*. We established a time course approach (measurements at 30, 60, 90, 180, 360, and 540 min post challenge) using cell-stable fluorescent dyes to measure ROS as well as specific generation of ${\text{O}}_{2}^{-}$ by BEA2B airway epithelial cells (**Figures 2A–D** and **Supplementary Figure 1**). We challenged both WT and ΔNlrx1 cell populations over 12 h with killed *A. fumigatus* conidia, killed hyphae, control buffer, or fungal PAMPs curdlan, zymosan, and zymosan depleted. Both curdlan and zymosan depleted are thought to be recognized by C-type lectin receptors such as Dectin-1, while zymosan is recognized by both human Tlr2 and Dectin-1. These three PAMPs are also commonly associated with the hyphal form of *A. fumigatus*.

Wild-type BEAS-2B cells produced ${\text{O}}_{2}^{-}$ within 30 min to the three hyphae-associated fungal PAMPs and killed hyphae, but not to killed conidia in comparison to control (**Figure 2A**). Concurrent measurements of ROS indicated a robust response to all five stimuli in comparison to control by 30 min, but diminished to or below baseline levels by 60–90 min, with the exception of zymosan, which remained elevated throughout (**Figure 2B**). ΔNlrx1 cells produced significantly decreased amounts of ${\text{O}}_{2}^{-}$ and general ROS in response to all stimuli in comparison to WT cells (*p* < 0.05) (**Figures 2C, D** and **Supplementary Figure 1**). The loss of ROS in the absence of Nlrx1 correlated well with a decrease in conidial processing observed during challenge assays (**Figure 1**). Our findings illuminate a very exciting phenomena that BEAS-2B airway cells do not produce detectable levels of ${\text{O}}_{2}^{-}$ over control PBS treatment in response to conidia, but do so for hyphae and hyphae-associated PAMPs. Further elevated ROS responses, which do occur for all five stimuli are mitigated in the absence of Nlrx1. The decrease in ROS in the absence of Nlrx1 fit well with the decreased processing of conidia observed by BEAS-2B cells.

We then conducted cellular ROS and OS measurements in BMDMs using killed conidia, and fungal PAMPs (**Figures 2E, F** and **Supplementary Figure 2**). Elevated and significant levels of ${\text{O}}_{2}^{-}$ and ROS were detected in response to all four stimuli within 30 min as expected for phagocytic cells (*p* < 0.05). Surprisingly, loss of Nlrx1 resulted in similar, non-statistically significant, levels of ${\text{O}}_{2}^{-}$ and ROS in comparison to wild-type (*p* > 0.10). This finding suggested that Nlrx1-dependent ${\text{O}}_{2}^{-}$ and ROS production does not meaningfully contribute to the diminished conidial processing observed for the absence of Nlrx1 in BMDMs.

We then performed similar experiments using BMDNs using the five stimuli. BMDNs did not produce elevated ${\text{O}}_{2}^{-}$ in response to killed conidia, but did to hyphae and hyphae-associated PAMPs (**Figures 2G, H** and **Supplementary Figure 3**). BMDNs did produce statistically significant levels of ROS in response to conidia (*p* < 0.05), but the amount was minuscule in comparison to killed hyphae and fungal PAMPs. Loss of Nlrx1 in BMDNs did not result in meaningful changes in ROS or OS production. Our initial assessment of ROS and OS regulation in response to *A. fumigatus* conidia and hyphae suggests that cell-type (BEAS-2B, BMDMs, and BMDNs) and morphotype (conidia, hyphae) responses are often, but not always, dependent upon Nlrx1 (summarized in **Table 1**).

**Table 1:** Summary of superoxide production and ROS production and their dependency on Nlrx1 in response to *A. fumigatus* and fungal PAMPs.

	BEAS-2B Airway Epithelial Cells			
	SuperoxideProduction	Nlrx1Dependency	ROSProduction	Nlrx1Dependency
**Conidia Killed**	**-**	**Not Applicable**	**+**	**Yes**
**Hyphae Killed**	**+**	**Yes**	**+**	**Yes**
**Curdlan**	**+**	**Yes**	**+**	**Yes**
**Zymosan**	**+**	**Yes**	**+**	**Yes**
**Zymosan Depleted**	**+**	**Yes**	**+**	**Yes**
	**Bone Marrow Derived Macrophages**			
	**Superoxide** **Production**	**Nlrx1** **Dependency**	**ROS** **Production**	**Nlrx1** **Dependency**
**Conidia Killed**	**+**	**No**	**+**	**No**
**Hyphae Killed**	**+**	**No**	**+**	**No**
**Curdlan**	**+**	**No**	**+**	**No**
**Zymosan**	**+**	**No**	**+**	**No**
**Zymosan Depleted**	**+**	**No**	**+**	**No**
	**Bone Marrow Derived Neutrophils**			
	**Superoxide** **Production**	**Nlrx1** **Dependency**	**ROS** **Production**	**Nlrx1** **Dependency**
**Conidia Killed**	**-**	**Not Applicable**	**+**	**No**
**Hyphae Killed**	**+**	**No**	**+**	**No**
**Curdlan**	**+**	**No**	**+**	**No**
**Zymosan**	**+**	**No**	**+**	**No**
**Zymosan Depleted**	**+**	**No**	**+**	**No**

Summarized data are from BEAS-2B airway epithelial cells, bone marrow-derived macrophages, and bone marrow-derived neutrophils.

### Loss of Nlrx1 Results in Diminished Mitochondrial Function in BEAS-2B Cells

**Figure 3:**
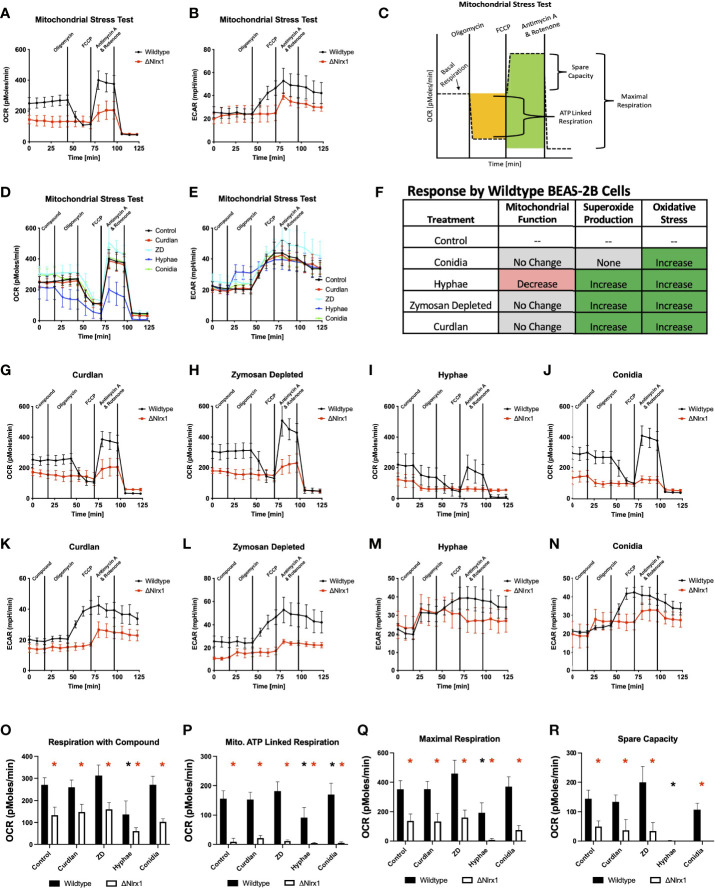
Modulation of mitochondrial energetics within wild-type BEAS-2B cells in response to *A. fumigatus* conidia, hyphae, and a subset of fungal PAMPs is dependent on Nlrx1. **(A)** Oxygen consumption rate (OCR, pmol/min) and **(B)** extracellular acidification rate (ECAR, mpH/min) for wild-type and Nlrx1-deficient (ΔNlrx1) cells using a standardized mitochondrial stress test outlined in **(C)**. Measurement of **(D)** OCR and **(E)** ECAR by wild-type BEAS-2B cells during a modified mitochondrial stress test. The modified version of the assay provides an injection of fungal PAMPs (zymosan depleted and curdlan), killed hyphae, or killed conidia prior to the standard test. **(F)** Summary of mitochondrial function, superoxide production, and general reactive oxygen species production by BEAS-2B cells in response to killed conidia, hyphae, and fungal PAMPs zymosan depleted, and curdlan. **(G–J)** OCR and **(K–N)** ECAR by wild-type and ΔNlrx1 BEAS-2B cells during a modified mitochondrial stress test using fungal PAMPs (zymosan depleted and curdlan), killed hyphae, or killed conidia. Calculated values for **(O)** respiration with compound, **(P)** mitochondrial ATP-linked respiration, **(Q)** maximal respiration, and **(R)** spare capacity. For **(O–R)**, black asterisk denotes *p* < 0.05 *via* Kruskal–Wallis test followed by a Dunn’s test for multiple comparisons against the control. Red asterisk denotes *p* < 0.05 between genotypes for a given treatment group.

Our results suggested that BEAS-2B cells were processing a significant amount of conidia in a manner that did not involve superoxide production, but correlated with the need to rapidly produce alternate forms of reactive oxygen species in a Nlrx1-dependent manner. ROS can be generated within the mitochondria as a product of oxidative phosphorylation (OxPhos) as well as in the cytoplasm by a variety of stress- and defense-related enzymes. To determine if Nlrx1 was contributing to mitochondrial ROS generation in BEAS-2B cells *via* OxPhos, we conducted standard and modified mitochondrial stress test using BEAS-2B cells pre-challenged with killed *A. fumigatus* conidia, hyphae, and fungal PAMPs curdlan, zymosan depleted, and zymosan (**Figure 3**).

Loss of Nlrx1 resulted in a near-complete loss of mitochondrial ATP-linked respiration, a significantly diminished maximal respiration and spare capacity using the standard mitochondrial stress test (*p* < 0.05, **Figures 3A–C, O–R**). Our data suggest that mitochondrial respiration is what we would assume to be near the minimum possible to retain viability for ΔNlrx1 BEAS-2B cells. At first impression, this would fit with the decreased production of ${\text{O}}_{2}^{-}$ and ROS. However, treatment of wild-type BEAS-2B cells with killed hyphae resulted in significantly decreased mitochondrial respiration, maximal respiration, and a complete lack of spare capacity (**Figures 3I, M, O–R**). This suggested that the observed increases in ROS and superoxide production in response to hyphae by WT BEAS-2B cells was not a part of mitochondrial OxPhos. Treatment of WT BEAS-2B cells with killed conidia or fungal PAMPs did not appear to modulate mitochondrial respiration, further indicating that the observed ${\text{O}}_{2}^{-}$ and general ROS responses did not correlate with changes in mitochondrial function (**Figures 3G, H, J–L, N–R**). Though the decrease in ROS and OS as well as mitochondrial function appear to correlate in the absence of Nlrx1, the significant decrease in mitochondrial function as measured by oxygen consumption by wild-type cells does not fit this hypothesis, suggesting that Nlrx1 function in ROS is occurring elsewhere or indirectly associated with its role in the mitochondria. Our results additionally highlight a novel finding that BEAS-2B cells respond to hyphae, but not conidia, by immediately diminishing mitochondrial OxPhos as measured by oxygen consumption. These unique responses fit the notion of morphotype-specific responses by the host cell that have been observed by our group and others.

### Glycolysis Is Differentially Modulated in Response to *A. fumigatus* Conidia and Hyphae in a Nlrx1-Dependent Manner

**Figure 4:**
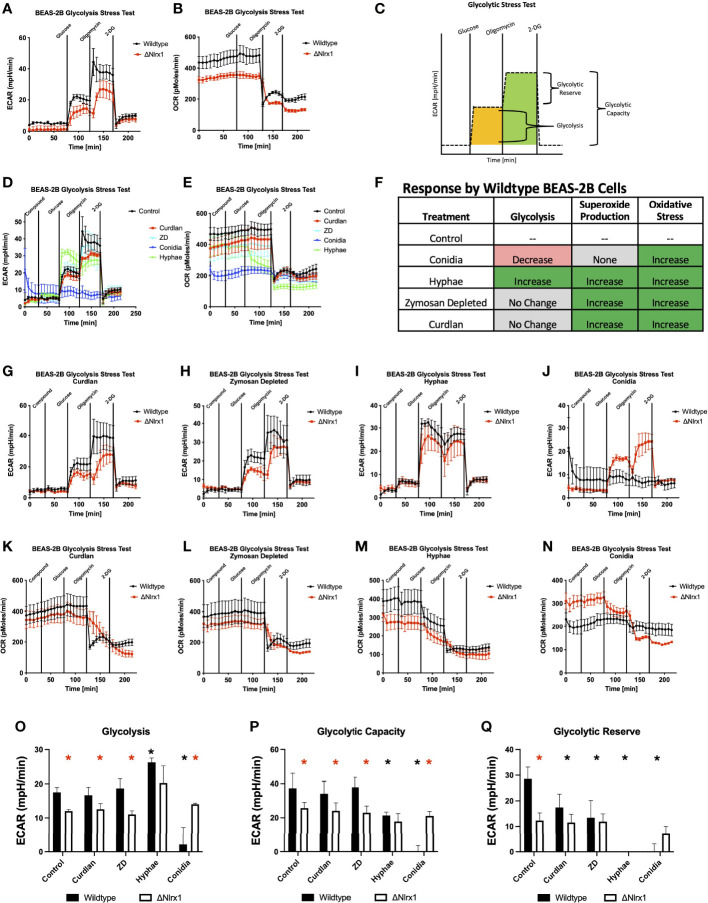
Modulation of glycolysis within BEAS-2B in response to *A. fumigatus* conidia, hyphae, and a subset of fungal PAMPs is dependent on Nlrx1. **(A)** Extracellular acidification rate (ECAR, mpH/min) and **(B)** oxygen consumption rate (OCR, pmol/min) for wild-type and Nlrx1-deficient (ΔNlrx1) cells using a standardized glycolytic stress test outlined in **(C)**. Measurement of **(D)** ECAR and **(E)** OCR by wild-type BEAS-2B cells during a modified glycolytic stress test. The modified version of the assay provides an injection of fungal PAMPs (zymosan depleted and curdlan), killed hyphae, or killed conidia prior to the standard test. **(F)** Summary of glycolysis, superoxide production, and general reactive oxygen species production by BEAS-2B cells in response to killed conidia, hyphae, and fungal PAMPs zymosan depleted and curdlan. **(G–J)** ECAR and **(K–N)** OCR by wild-type and ΔNlrx1 BEAS-2B cells during a modified glycolytic stress test using fungal PAMPs (zymosan depleted and curdlan), killed hyphae, or killed conidia. Calculated values for **(O)** glycolysis, **(P)** glycolytic capacity, and **(Q)** glycolytic reserve. For **(O–Q)**, black asterisk denotes *p* < 0.05 *via* Kruskal–Wallis test followed by a Dunn’s test for multiple comparisons against the control. Red asterisk denotes *p* < 0.05 between genotypes for a given treatment group.

Given the novel observation of decreased mitochondrial respiration in response to hyphae and the lack of direct correlation between mitochondrial respiration and superoxide/ROS production in response to *A. fumigatus* by BEAS-2B cells, we thought to explore if glycolysis is perturbed in response to *A. fumigatus* and if this process is dependent on Nlrx1. Early aspects of glycolysis serve as a central conduit to the pentose phosphate pathway, which is a critical source of NADPH utilized by NADPH oxidases to generate ROS. When sugars are diverted to the PPP, glycolysis can also become effectively diminished. Similarly, movement of molecules into the TCA cycle can give a false appearance that glycolysis is shut down when in fact glycolysis is occurring just not *via* lactate production as measured in the glycolytic stress test. We conducted a standard glycolytic stress test as well as the modified form where we once again pretreated cells with killed *A. fumigatus* conidia, hyphae, and fungal PAMPs. The basal glycolytic stress test indicated that Nlrx1-deficient cells were statistically deficient in glycolysis and had diminished glycolytic capacity and glycolytic reserve (*p* < 0.05) (**Figures 4A–C, O–Q**). Thus, the absence of Nlrx1 also decreases basal glycolysis function and reserve.

Surprisingly, pre-treating BEAS-2B cells with killed conidia resulted in near-complete shutdown of glycolysis, glycolytic capacity, and reserve during the glycolytic stress test. This highly novel and significant finding suggests that glycolysis is shut down in response to killed conidia (**Figures 4D, E**). This shutdown may be part of a process to shunt glucose into the PPP to facilitate NADPH production to be utilized for ROS production, thereby reducing lactate production, which is indirectly measured *via* decreased changes in pH over time (ECAR) by the Seahorse metabolic flux analyzer. Even more surprising, Nlrx1-deficient BEAS-2B cells did not shut down glycolysis in response to conidia and had a similar response to Nlrx1-deficient cells treated with control buffer (**Figures 4J, N–Q**). We conclude that loss of Nlrx1 results in a failure to shut down glycolysis in response to conidia by BEAS-2B cells. This failure to shut down glycolysis may provide an explanation into why enhanced conidial germination and decreased ROS production is observed for ΔNlrx1 cells.

Contrastingly, treatment of BEAS-2B cells with killed hyphae prior to the stress test indicated significantly elevated glycolysis, decreased glycolytic capacity, and a near-complete depletion of the glycolytic reserve in comparison to control cells (*p* < 0.05) (**Figures 4D, E, I, M, O–Q**). Nlrx1-deficient BEAS-2B cells treated with hyphae were also elevated in glycolysis in comparison to control treatments yet were decreased in glycolysis activation in comparison to wild type treated with hyphae. Both WT and Nlrx1-deficient BEAS-2B cells were completely depleted of any glycolytic reserves, suggesting significant bioenergetics in response to stimuli.

Treatment with curdlan and zymosan did not significantly elevate glycolysis, but did significantly decrease glycolytic reserve in comparison to control BEAS-2B cells (**Figures 4G, H, K, L**). Ultimately, our results suggest that BEAS-2B cells activate glycolysis and deplete reserves in response to killed hyphae while turning off glycolysis in response to conidia. Nlrx1-deficient cells fail to ablate glycolysis in response to conidia and can only partially elevate glycolysis in response to hyphae (**Figure 4F**).

### Block-Aid of Pentose Phosphate Pathway Reduces ROS Production and Processing in Response to *A. fumigatus* Conidia

Our results from the modified glycolytic stress tests indicated that glycolysis is significantly diminished in response to conidia and is also controlled *via* a Nlrx1-dependent process or potentially Nlrx1 directly. This finding fit well with the observation of decreased ROS production by ΔNlrx1 BEAS-2B cells and enhanced conidial germination. We hypothesized that the shutdown of glycolysis was occurring to facilitate a movement of energy towards the PPP to generate NADPH. To test this hypothesis, we treated wild-type BEAS-2B cells with 2-DG, an inhibitor of hexokinase and glucose-6-phosphate isomerase, thereby inhibiting glycolysis and PPP, as well as glucose-6-phosphate dehydrogenase inhibitor 1, an inhibitor of the enzyme responsible for NADPH production in PPP. We also tested sodium oxamate, an inhibitor of lactate dehydrogenase, and a combination of Rotenone/Antimycin A, potent inhibitors of mitochondrial complex I and III, respectively.

**Figure 5:**
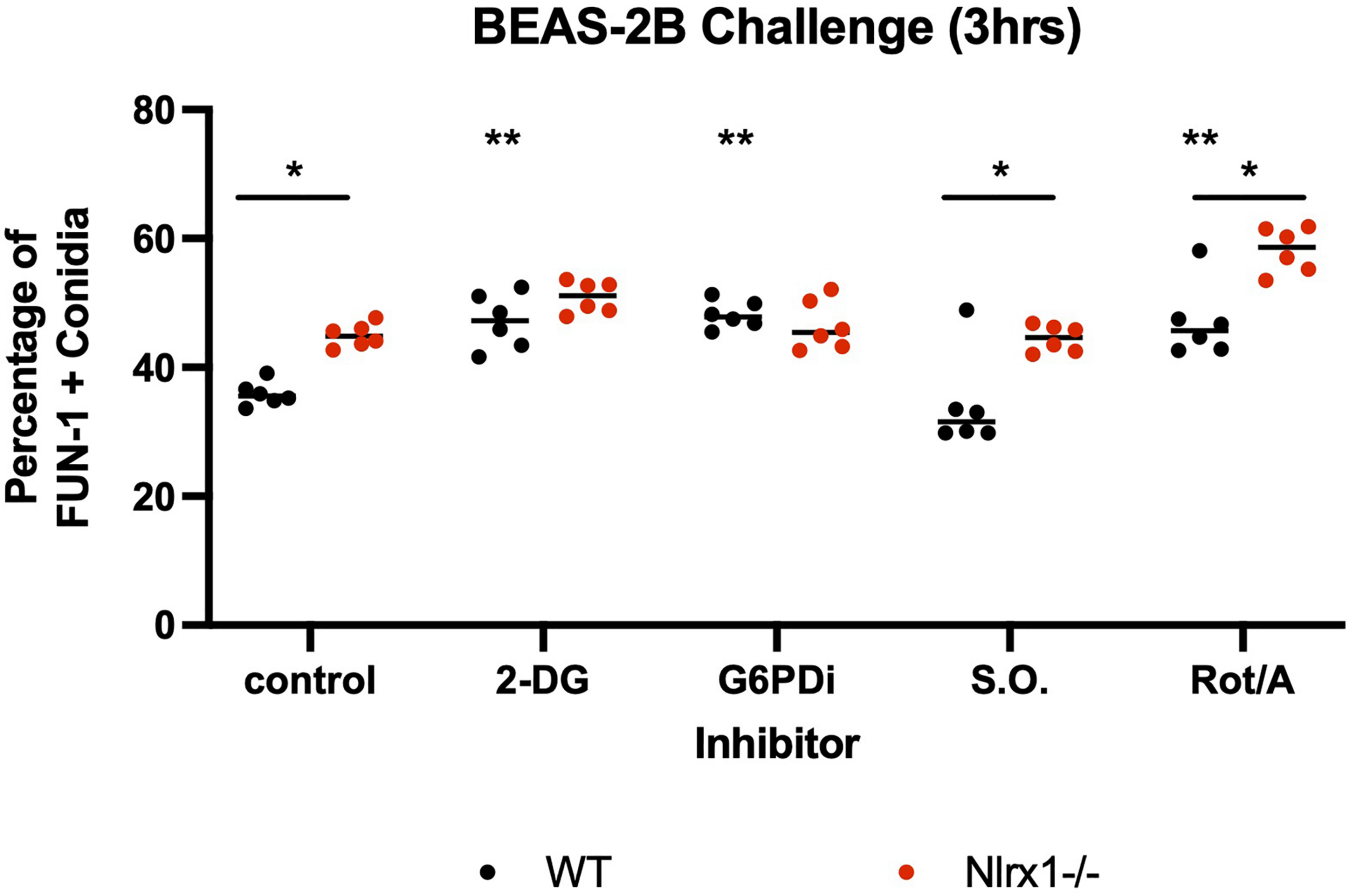
Inhibition of glycolysis and mitochondrial function in BEAS-2B reduces early conidial processing. Wild-type and Nlrx1-deficient (ΔNlrx1) BEAS-2B cells were pre-incubated with inhibitors at varying concentrations for 3 h. Cells were then washed three times with 2 × PBS to remove all inhibitors. Cells were then challenged with AF293 for 3 h and a FUN-1+ fluorescence assay was subsequently conducted. Final concentration of inhibitors: 2-DG, 25 mM; G6PDi-1, 50 μM; Sodium oxamate (S.O.), 25 mM; Rotenone/Antimycin A (Rot/Ant), 0.5 μM. Black double asterisk denotes *p* < 0.05 *via* Kruskal–Wallis test followed by a Dunn’s test for multiple comparisons against the control. Black single asterisk with a line across the two genotypes denotes *p* < 0.05 between genotypes for a given treatment group.

**Figure 6:**
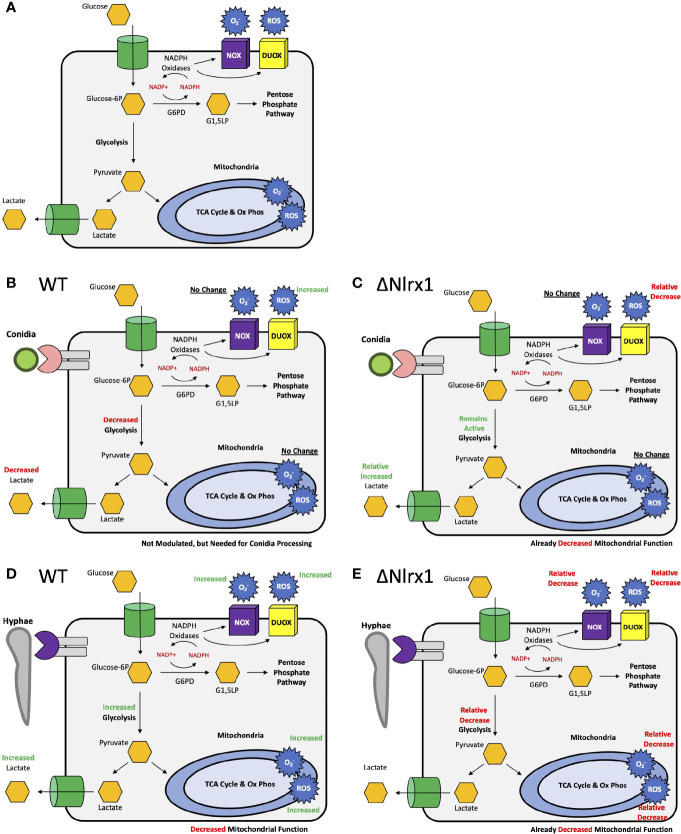
Simplified, working models for metabolic responses to conidia and hyphae in airway epithelial cells and their dependency on Nlrx1. **(A)** Simplified representation of glycolysis, the pentose phosphate pathway, oxidative phosphorylation, ROS, and superoxide production. Modulation of these pathway in response to **(B)** conidia and **(D)** hyphae by wild-type cells. Relative modulation of these pathways in response to **(C)** conidia and **(E)** hyphae by Nlrx1-deficient cells in comparison to wild type.

Pre-treatment of wild-type BEAS-2B cells with either 2-DG or glucose-6-phosphate dehydrogenase inhibitor 1 resulted in a significantly increased percentage of metabolically viable conidia in comparison to control and produced similar viability levels as ΔNlrx (*p* < 0.05, **Figure 5**). Pre-treatment with sodium oxamate, a specific inhibitor of lactate dehydrogenase, did not alter conidial processing for WT or ΔNlrx1 cells (*p* > 0.10), while the treatment with Rot/AntA resulted in a statistically significant increase in conidial survival for both genotypes (*p* < 0.05, **Figure 5**). These results fit well with our working model that glycolysis is shut down in order to shunt glucose 6-phosphate to fuel the pentose phosphate pathway specifically NADPH production. While our metabolic analysis *via* Seahorse metabolic flux analyzer did not identify a perturbation in mitochondrial function in response to killed conidia. Our inhibitor challenge assays suggest that loss of basal mitochondria function results in a Nlrx1-independent decrease in conidial viability. A summary, working model is presented in **Figure 6** for how BEAS-2B cells respond to conidia and hyphae in a Nlrx1-dependent manner based on our findings.

## Discussion

Host resistance and clearance of *A. fumigatus*, and other ubiquitous microorganisms inhaled into the respiratory system, is an inherent and evolved feature with significant selection pressure. Mucociliary clearance is thought to be a key mechanism by which conidia and fungal particulate are removed from the upper respiratory system. Beyond mucociliary clearance, there exists a highly coordinated process of inter- and intracellular signaling events leading to the appropriate recognition, processing, containment, and eventual clearance of *A. fumigatus*. Aberrations in these processes may result in a variety of disease manifestation. Our prior results using Nlrx1-deficient mice in models of IPA indicated enhanced immunopathogenesis due to loss of regulation in both inter- and intracellular signaling as well as fungus burden, thereby resulting in a double detriment. Our *in vitro* studies indicate a defect in processing and killing of conidia by Nlrx1-deficient airway epithelial cells, alveolar macrophages, and BMDMs. This fit well with the enhanced fungal burden observed for Nlrx1-deficient mice in our prior studies on day 1 and day 3 post challenge using three different models of IPA and one of immuno-competent challenge (6).

Our results presented in this study indicate that conidia do not induce a specific production of superoxide in BEAS-2B cells, but rather induce general ROS production as an early burst that may be associated with general oxidative stress or specific production for defense purposes. The intensity of this burst was significantly lower in ΔNlrx1 cells. Reactive oxygen species can be produced as either a product or by-product of mitochondrial function during oxidative phosphorylation (33). ROS, specifically hydrogen peroxide, can also be produced *via* NADPH-dependent DUOXs (DUOX1 and DUOX2) in a non-phagocytic manner at the cell membrane (34). Given our prior work and that of others on extracellular processing of conidia by airway epithelial cells (11, 18), DUOXs are a prime candidate for this function. DUOXs have also been previously shown to be critical in the killing and/or immune response to a number of microbes including *Helicobacter felix, Psuedomonas aeruginosa, Enterococcus faecalis, Candida albicans*, and influenza A virus, as well as maintaining gut and pulmonary homeostasis (35–41). Given the lack of mitochondrial perturbation in response to conidia as well as the Nlrx1-independent effect of Rot/AntA on conidial processing, we suspect that the Nlrx1-dependent ROS burst observed would therefore be due to NADPH oxidases such as DUOXs.

Cellular NADPH can be generated from glucose-6-phosphate dehydrogenase (G6PD) in the pentose phosphate pathway, cytoplasmic isocitrate dehydrogenase (IDH1), and cytoplasmic maleic enzyme (ME1) [Reviewed in (42)]. Our analysis of glycolysis *via* Seahorse metabolic flux analyzer indicated that glycolysis was significantly decreased in response to conidia by wild-type BEAS-2B cells. One explanation to this occurrence was the need to move molecules into the pentose phosphate pathway. Inhibition of glycolysis and the pentose phosphate pathway *via* 2-DG and the specific inhibition of G6PD *via* G6PDi resulted in significant increase in conidial survival post challenge against WT BEAS-2B cells to levels similar to the Nlrx1 knockout. Our results in total suggest that the shutdown of glycolysis in response to *A. fumigatus* conidia is regulated by Nlrx1 to shunt glucose towards the pentose phosphate pathway to generate NADPH. This response has not been observed for *A. fumigatus* conidia or clinically relevant fungi to the best of our knowledge. How Nlrx1 shuts down glycolysis in response to *A. fumigatus* conidia is of high relevance as this cellular metabolic function is critical for processing conidia. The increase in glycolysis and depletion of reserves in response to hyphae suggest that metabolism is an important component in mediating the appropriate morphotype-specific response to *A. fumigatus*.

An increase in glycolysis in response to a bacteria, parasites, and viruses has been documented for several pathosystems (43–46). A recent study has observed enhanced glycolysis in response to *A. fumigatus* conidia by macrophage cell types (8). Our results support this connection as we noted a robust production of superoxide and ROS by BMDMs that is Nlrx1 dependent. Differences in the method of ROS generation between the cell types will be exciting to explore into the future as ample prior evidence suggests cell-specific ROS generation [reviewed in (34)]. Similarly, our work also suggests that Nlrx1 plays a critical role in not only shutting down glycolysis when needed in response to conidia, but also its amplification in response to hyphae. How this contrasting modulation of glycolysis occurs will provide unique insight into the underlying differences for these signaling pathways in different cell types.

Much of our current study has centered around the role of an early ROS burst in response to *A. fumigatus* that is dependent on Nlrx1. However, it is critical to acknowledge a number of additional non-oxidative defense strategies such as secreted peptides and proteins (47–49) may also be differentially expressed in a decreasing manner in the Nlrx1-deficient background. One broad conclusion of our study is that the functions and roles of Nlrx1 continue to extend, and these processes, such as an absence of negative regulation of glycolysis may also impact additional processes within the cell such as gene expression of defense proteins.

Our preliminary work with BMDMs and alveolar macrophages indicated that Nlrx1 is critical for conidial processing. Macrophages rapidly internalize *A. fumigatus* conidia and process conidia in a manner dependent on NADPH oxidases as shown through p47phox deficient macrophages and chemical inhibitors of NADPH oxidases (10). P47phox functions as a central organizer of the NOX2 NADPH oxidase phagocytic complex that produces superoxide (10). We observed small decreases in superoxide and ROS production by Nlrx1-deficient BMDMs indicating that Nlrx1 may be modulating additional non-oxidative aspects of conidial processing. Nlrx1 has been shown to facilitate LC3-associated phagocytosis (LAP) of *Histoplasma capsulatum* in macrophages *via* cytoplasmic interactions with TUFM/ATG5-ATG12, but not Dectin-1/Syk-mediated ROS production (50). Loss of Nlrx1 did not impact *H. capsulatum* replication, only LAP. It will be interesting to explore if Nlrx1 regulates phagocytosis of *A. fumigatus* conidia in macrophages and neutrophils and if this process has an impact on conidial processing.

There have been a variety of reports indicating ROS production being independent or dependent on Nlrx1. Overexpression and targeting of NLRX1 to the mitochondria triggered ROS production to levels similar to that produced by TNFα treatment in HeLa cells, suggesting a dependence on Nlrx1 (23). Concurrent overexpression of Nlrx1 and treatment of HeLa cells with either TNFα, *Shigella* infection, or the viral RNA analog polyinosinic:polycytidylic acid resulted in further enhanced ROS production (23). Similarly, Nlrx1 overexpression was shown to enhance *Chlamydia trachomatis-*induced ROS production in HeLa cells (24). This ROS production could be inhibited *via* either DPI treatment, indicating that the source was cytoplasmic or membrane NOX and/or DUOX proteins or by siRNA targeting either DUOX1, DUOX2, or NOX4. This finding fits well with our hypothesis that Nlrx1 is regulating ROS production external to the mitochondria in BEAS-2B epithelial cells.

Conversely, loss of Nlrx1 has been shown to increase ROS production by BMDMs in response to *H. pylori* that correlated with decreased *H. pylori* survival post challenge (25). In animal and cell models of ischemia–reperfusion injury reactive oxygen species, specifically mitochondrial superoxide production was shown to increase in the absence of Nlrx1 (51). No statistically significant or minimal decrease in ROS production was observed in *Nlrx1-/-* BMDMs and neutrophils in comparison to WT with a variety of PAMPs (LPS, TNFα, polyI:C, Pam3Cys, and fmlp) (28). Our results were in accord with these finding as we did not observe a Nlrx1 dependency on superoxide or ROS production by BMDMs and BMDNs in response to fungal PAMPs or killed conidia. Relatedly, Nlrx1 was shown to not influence Syk/NADPH mediated ROS production in response to fungal pathogen *Histoplasma capsulatum*, but Nlrx1 did modulate LC3 phagocytosis by macrophages *via* cytoplasmic interactions with TUFM/ATG5-ATG12 (50). In totality, the cell-type and morphotype specificity to *A. fumigatus* and fungal PAMPs observed in our work transcends to a wider scope and implies specific pathway level regulation of ROS production that is dependent on cell type and stimuli.

The function of Nlrx1 in mitochondrial respiration has also been contrasting among studies, and recent meta-analysis suggests a highly cell-type-specific functionality of Nlrx1 (52). Mechanistically, Nlrx1 has been shown to translocate into the mitochondria where it interacts with UQCRC2, a member of complex III, and Fas-activated serine-threonine kinase family protein-5 (FASTKD5) (22, 23, 53). The Nlrx1/UQCRC2/Complex III is thought to impact mitochondrial ROS as well as mitochondrial respiration (22, 23, 53). Nlrx1 binding of FASTKD5 and mitochondrial RNA leads to an inhibition of maturation for transcripts encoding complex I and IV components (54). Loss of Nlrx1 results in an increased rate of OxPhos in hepatocytes presumably due to maturation of these transcripts (54). In epithelial cell (HEK293) models of ischemia–reperfusion injury, loss of Nlrx1 also resulted in increased oxygen consumption, oxidative stress, and downstream apoptosis (51). In CD4+ T cells, activation of Nlrx1 leads to increased expression of markers associated with oxidative phosphorylation (29). Our results with BEAS-2B epithelial cells indicate that loss of Nlrx1 resulted in decreased oxygen consumption, mitochondrial function, spare capacity, and maximal respiration during the mitochondrial stress test. We also observed a decreased oxygen consumption during the glycolytic stress test. Ultimately, these findings add the notion of Nlrx1 being highly cell type specific in regards to mitochondrial function.

While our hypothesis is centered on the loss of Nlrx1 resulting in a failure to modulate pathways at the protein level, an interesting hypothesis is that the loss of Nlrx1 has resulted in an underlying change in gene expression that results in the loss of signal transduction pathway components from being expressed versus a failure for that pathway to transduce that signal in the absence of Nlrx1. Given the observed effects of Nlrx1 on maturing complex I and IV components as well as gene expression of OxPhos components, this will be an important consideration in the future (29, 54). Ongoing studies will allow us to determine if loss of Nlrx1 changes the underlying transcriptome.

The interplay between host metabolism, immune signaling, and cellular defense towards microbes is an exciting field of study that presents novel therapeutic options and insight into host biology. One aspect of the intertwined and complex nature of these processes has been partially illuminated in the context of Nlrx1. Why and how BEAS-2B cells decrease glycolysis in response to conidia yet increase for hyphae will be an exciting exploration for future studies. Furthermore, the physiological relevance of why glycolysis is decreased by non-hematopoietic stem cell populations such as airway epithelial cells in response to conidia will also be important to determine.

## Data Availability Statement

The raw data supporting the conclusions of this article will be made available by the authors, without undue reservation.

## Ethics Statement

The animal study was reviewed and approved by the NIMML Institute.

## Author Contributions

SK conceived the idea. SK, JB-R, and RH designed the experiments. BK, TA, NT-J, and SK conducted the experiments. BK, TA, NT-J, AL, and SK analyzed the results. BK and SK wrote the manuscript with input from all authors. All authors contributed to the article and approved the submitted version.

## Conflict of Interest

The authors declare that the research was conducted in the absence of any commercial or financial relationships that could be construed as a potential conflict of interest.

## Publisher’s Note

All claims expressed in this article are solely those of the authors and do not necessarily represent those of their affiliated organizations, or those of the publisher, the editors and the reviewers. Any product that may be evaluated in this article, or claim that may be made by its manufacturer, is not guaranteed or endorsed by the publisher.

## Supplementary Material

Supplementary Figure 1 Reactive oxygen species and oxidative stress production by wild type and ΔNlrx1 BEAS-2B airway epithelial in response to fungal PAMPs and *A. fumigatus*. Wild type and ΔNlrx1 BEAS-2B cells were pre-incubated with **(A–F)** a fluorescent dye specific for superoxide production or **(G–L)** a fluorescent oxidative stress (OS) indicator. Cells were then treated with **(A, G)** control PBS buffer, **(B, H)** killed hyphae, **(C, I)** zymosan, **(D, J)** curdlan, **(E, K)** zymosan depleted, or **(F, L)** killed conidia for 9hrs. Fluorescence was measured at 30, 60, 90, 180, 360, 540 min post treatment. N=6-8. Error bars denote standard deviation. All experiments were independently repeated. *P* < 0.05, indicated by asterisk *via* Kruskal-Wallis test followed up by a Dunn’s test for multiple comparisons.

Supplementary Figure 2 Reactive oxygen species and oxidative stress production by wild type and Nlrx1 deficient bone marrow derived macrophages in response to fungal PAMPs and *A. fumigatus*. Wild type and Nlrx1 deficient bone marrow derived macrophages (BMDMs) were pre-incubated with **(A–F)** a fluorescent dye specific for superoxide production or **(G–L)** a fluorescent oxidative stress (OS) indicator. Production of **(A)** superoxide and **(B)** reactive oxygen species by wildtype BMDMs to stimuli (killed conidia, zymosan depleted, zymosan, curdlan, and control PBS) over 9 hours. Wildtype and Nlrx1-/- BEAS-2B cells were then treated with either **(B, H)** curdlan, **(C, I)** zymosan depleted, **(D, J)** zymosan, **(E, K)** killed conidia or **(F, L)** control PBS buffer over 9hrs. Fluorescence was measured at 30, 60, 90, 180, 360, 540 min post treatment. N=8. Error bars denote standard deviation. All experiments were independently repeated. *P* < 0.05, indicated by asterisk *via* Kruskal-Wallis test followed by a Dunn’s test for multiple comparisons.

Supplementary Figure 3 Reactive oxygen species and oxidative stress production by wild type and Nlrx1 deficient bone marrow derived neutrophils in response to fungal PAMPs and *A. fumigatus*. Wild type and Nlrx1 deficient bone marrow derived neutrophils (BMDNs) were pre-incubated with **(A–G)** a fluorescent dye specific for superoxide production or **(H–N)** a fluorescent oxidative stress (OS) indicator. Production of **(A, B)** superoxide and **(H, I)** reactive oxygen species by wildtype BMDNs to stimuli (killed conidia, zymosan depleted, zymosan, curdlan, and control PBS) over 6 hours. Cells were then treated with **(C, J)** curdlan, **(D, K)** zymosan depleted, **(E, L)** zymosan, **(F, M)** killed conidia or **(G, N)** control PBS buffer for 6hrs. Fluorescence was measured at 30, 60, 90, 180, 360 min post treatment. N=8. Error bars denote standard deviation. All experiments were independently repeated. *P* < 0.05, indicated by asterisk *via* Kruskal-Wallis test followed by a Dunn’s test for multiple comparisons.
