# Supplementary figures and images for: Nlrx1-Regulated Defense and Metabolic Responses to Aspergillus fumigatus Are Morphotype and Cell Type Specific
Source: Front Immunol. 2021 Nov 1;12:749504. doi: 10.3389/fimmu.2021.749504 (PMC8591139; doi:10.3389/fimmu.2021.749504)

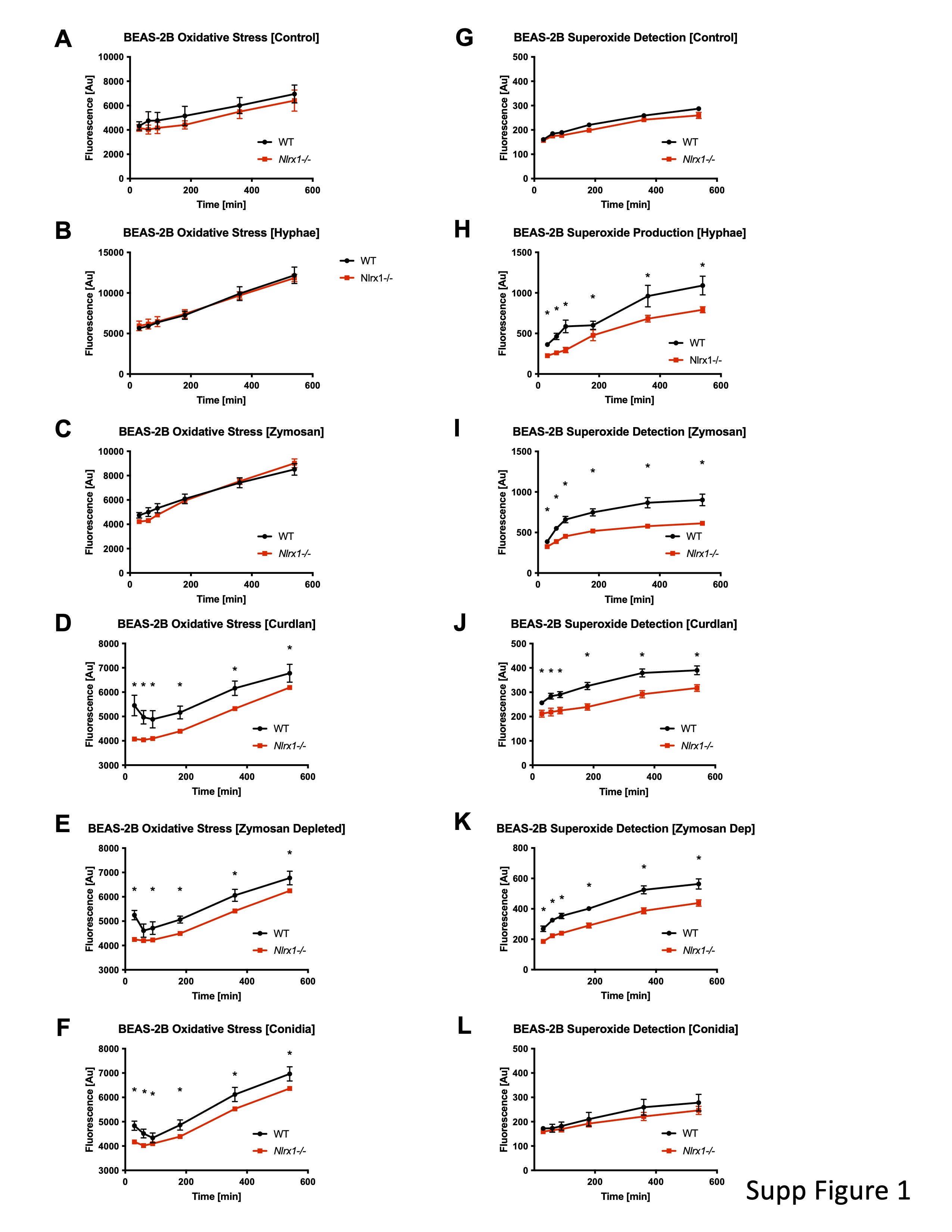

Supplement: Supplementary Figure 1 — Reactive oxygen species and oxidative stress production by wild type and ΔNlrx1 BEAS-2B airway epithelial in response to fungal PAMPs and A. fumigatus. Wild type and ΔNlrx1 BEAS-2B cells were pre-incubated with (A–F) a fluorescent dye specific for superoxide production or (G–L) a fluorescent oxidative stress (OS) indicator. Cells were then treated with (A, G) control PBS buffer, (B, H) killed hyphae, (C, I) zymosan, (D, J) curdlan, (E, K) zymosan depleted, or (F, L) killed conidia for 9hrs. Fluorescence was measured at 30, 60, 90, 180, 360, 540 min post treatment. N=6-8. Error bars denote standard deviation. All experiments were independently repeated. P < 0.05, indicated by asterisk via Kruskal-Wallis test followed up by a Dunn’s test for multiple comparisons. [file Image_1.jpeg]

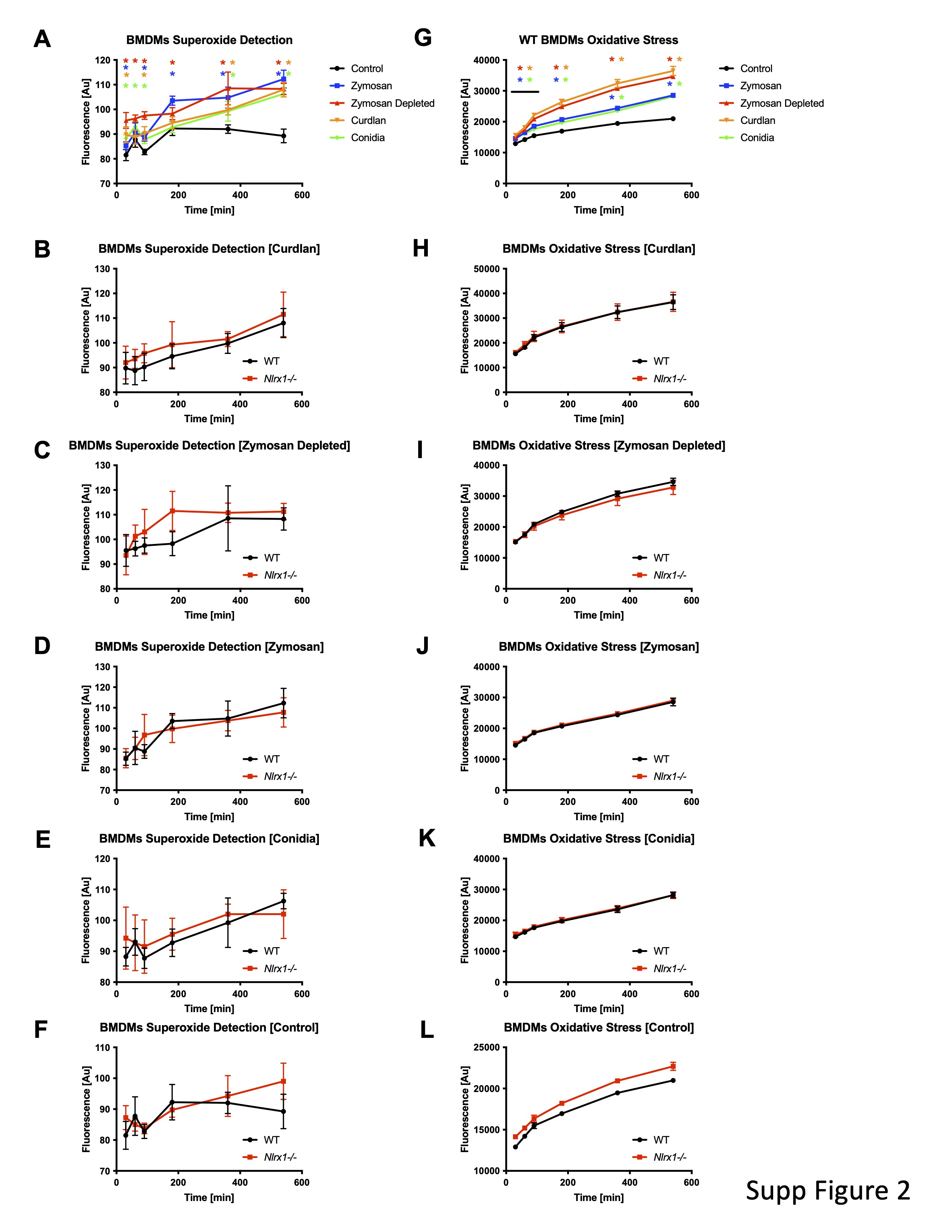

Supplement: Supplementary Figure 2 — Reactive oxygen species and oxidative stress production by wild type and Nlrx1 deficient bone marrow derived macrophages in response to fungal PAMPs and A. fumigatus. Wild type and Nlrx1 deficient bone marrow derived macrophages (BMDMs) were pre-incubated with (A–F) a fluorescent dye specific for superoxide production or (G–L) a fluorescent oxidative stress (OS) indicator. Production of (A) superoxide and (B) reactive oxygen species by wildtype BMDMs to stimuli (killed conidia, zymosan depleted, zymosan, curdlan, and control PBS) over 9 hours. Wildtype and Nlrx1-/- BEAS-2B cells were then treated with either (B, H) curdlan, (C, I) zymosan depleted, (D, J) zymosan, (E, K) killed conidia or (F, L) control PBS buffer over 9hrs. Fluorescence was measured at 30, 60, 90, 180, 360, 540 min post treatment. N=8. Error bars denote standard deviation. All experiments were independently repeated. P < 0.05, indicated by asterisk via Kruskal-Wallis test followed by a Dunn’s test for multiple comparisons. [file Image_2.jpeg]

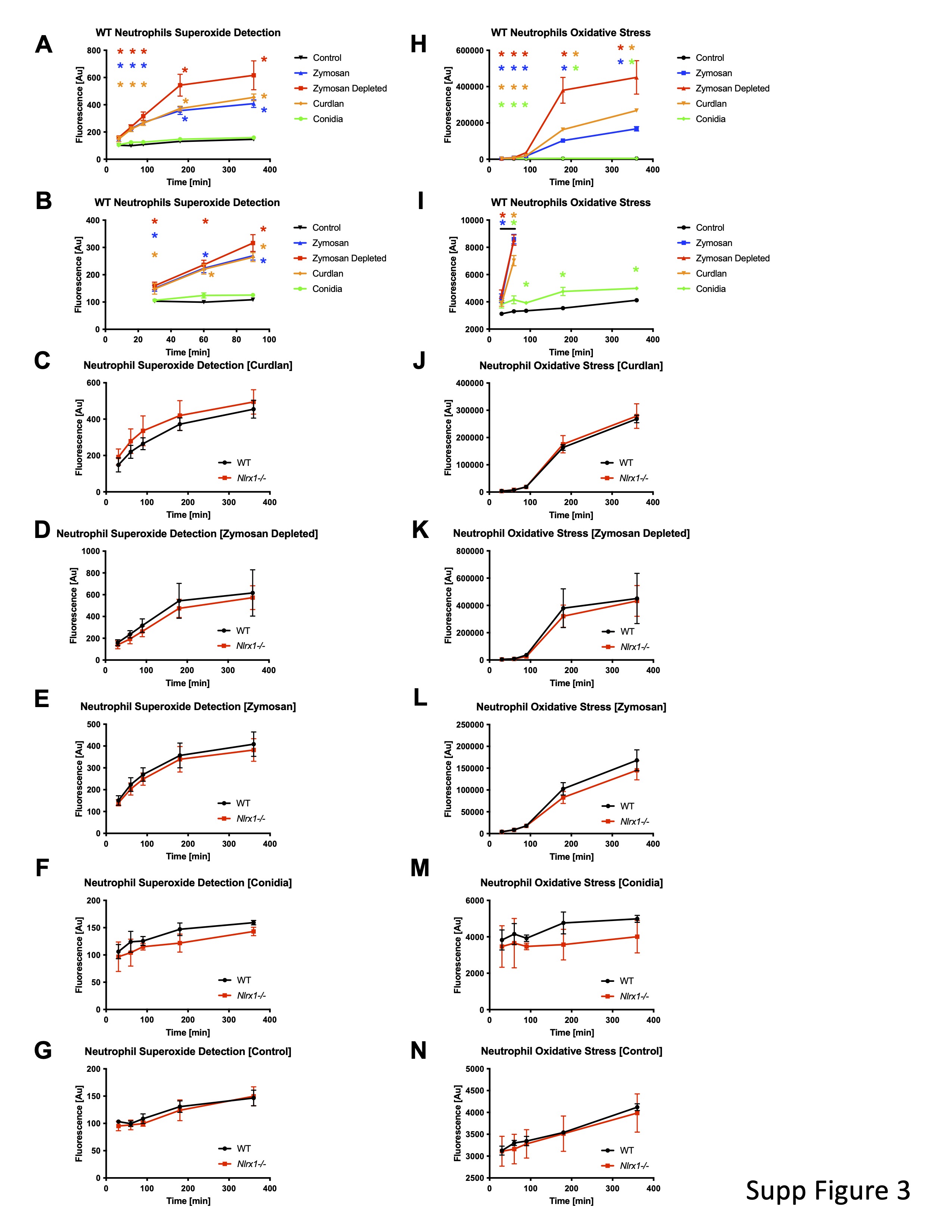

Supplement: Supplementary Figure 3 — Reactive oxygen species and oxidative stress production by wild type and Nlrx1 deficient bone marrow derived neutrophils in response to fungal PAMPs and A. fumigatus. Wild type and Nlrx1 deficient bone marrow derived neutrophils (BMDNs) were pre-incubated with (A–G) a fluorescent dye specific for superoxide production or (H–N) a fluorescent oxidative stress (OS) indicator. Production of (A, B) superoxide and (H, I) reactive oxygen species by wildtype BMDNs to stimuli (killed conidia, zymosan depleted, zymosan, curdlan, and control PBS) over 6 hours. Cells were then treated with (C, J) curdlan, (D, K) zymosan depleted, (E, L) zymosan, (F, M) killed conidia or (G, N) control PBS buffer for 6hrs. Fluorescence was measured at 30, 60, 90, 180, 360 min post treatment. N=8. Error bars denote standard deviation. All experiments were independently repeated. P < 0.05, indicated by asterisk via Kruskal-Wallis test followed by a Dunn’s test for multiple comparisons. [file Image_3.jpeg]
